# Potassium-rich mining waste addition can shorten the composting period by increasing the abundance of thermophilic bacteria during high-temperature periods

**DOI:** 10.1038/s41598-023-31689-3

**Published:** 2023-04-13

**Authors:** Xiao-jun Huo, YanZhou, Min-jie Chen, Jian-lin Zhou, Chun-li Zheng

**Affiliations:** 1 Inner Mongolia Research Academy of Eco-Environmental Sciences, Hohhot, 010000 Inner Mongolia China; 2 School of Resources and Environmental Engineering, Shanghai Polytechnic University, Shanghai, 201209 Shang Hai China; 3 School of Life Science and Technology, Inner Mongolia University of Science and Technology, Baotou, 014010 Inner Mongolia China; 4 Engineering Research Center of Evaluation and Restoration in the Mining Ecological Environments, Inner Mongolia University of Science and& Technology, Baotou, 014010 Inner Mongolia China

**Keywords:** Microbiology, Environmental social sciences, Energy science and technology

## Abstract

Conventional compost sludge has a long fermentation period and is not nutrient rich. Potassium-rich mining waste was used as an additive for aerobic composting of activated sludge to make a new sludge product. The effects of different feeding ratios of potassium-rich mining waste and activated sludge on the physicochemical properties and thermophilic bacterial community structure during aerobic composting were investigated. The results showed that potassium-rich waste minerals contribute to the increase in mineral element contents; although the addition of potassium-rich waste minerals affected the peak temperature and duration of composting, the more sufficient oxygen content promoted the growth of thermophilic bacteria and thus shortened the overall composting period. Considering the requirements of composting temperature, it is recommended that the addition of potassium-rich waste minerals is less than or equal to 20%.

## Introduction

Approximately 5,476 urban wastewater treatment plants were in operation in China by the end of 2019, and the production of domestic sludge reached 39.04 million tons. Among the many utilization techniques, land use accounted for the highest percentage, accounting for approximately 29.3% of all the ways of utilization^1^. Sludge land use requires environmentally sound treatment before it can be used. Composting is a technology that can effectively reduce or eliminate pathogens and organic contaminants from sludge^2^^,^^3^. Composting has the advantages of environmental friendliness, low cost and high social acceptability. At the same time, the choice of composting materials is flexible, so a joint composting scheme can be designed according to composting needs and waste characteristics to improve composting quality^4^. Composting is receiving increasing attention^5^. The choice of composting material is mostly organic waste, including animal manure^6^, sewage sludge^7^, plant residues or agricultural waste^8^. Some additives act as bulking agents, such as biochar^9^, rice husk, straw, and eggshell^10^. Bulking agents can shorten the composting period to a large extent, and the reasons have mostly been reported as improved gas exchange in the compost pile and accelerated compost microbial succession. Some studies^11^ proposed the hypothesis that a high concentration of oxygen can shorten the composting period and confirmed the important relationship between the oxygen content and the composting cycle by electrolyzing water to produce oxygen. However, the cost of this oxygen supply method is too high to achieve industrial penetration at this time. Therefore, the transition still needs to be carried out through the improvement of materials.

In recent years, studies on composting with the addition of inorganic materials have been reported^12^^,^^13^. A reasonable combination of organic and inorganic waste can optimize the composting process and improve product quality. This idea provides new research directions for aerobic composting experiments with the addition of minerals for nutrient regulation. For example, the addition of phosphogypsum in the composting process can effectively improve the porosity and bulk weight of the pile^14^. Potassium feldspar is known for its high potassium content and prevalence in the Earth’s crust^15^. However, potassium feldspar usually contains a large number of other elements that can promote plant growth, such as silicon^16^, magnesium^17^ and calcium^18^, which also play an important role in plant nutrient regulation. However, it is considered a kind of potassium-rich mining waste because of its insolubility. If potassium-rich mining waste such as potash feldspar is used in conventional sludge compost, it will not only enrich the mineral elements of traditional sludge fertilizer but also consume a large amount of potassium-rich mining waste.

Composting relies heavily on the metabolic action of microorganisms. Microorganisms directly determine the composting process and product quality. Adding an appropriate amount of potassium-rich mining waste may be an effective way to optimize the composting process and promote the utilization of composting products, but this has not been proven at the microbial level. The effect of mineral composition on the thermophilic bacteria of aerobic composting has rarely been discussed in previous studies. Therefore, it is necessary to investigate the key microorganisms of potassium-rich mining waste composting. In this study, we analyzed the thermophilic bacterial community structure and functional information by using high-throughput sequencing technology. The objectives of this report were to (1) investigate the effects of different potassium-rich mining waste and sludge ratios on composting physicochemical properties and thermophilic bacterial community structure; (2) test the hypothesis that potassium-rich waste minerals promote the growth of thermophilic bacteria by improving the internal environment of the compost, thereby shortening the composting time; and (3) determine the most efficient addition of potassium-rich mining waste and to provide a low-cost, efficient and qualified composting solution for potassium-rich mining waste.

## Materials and methods

### Compost fermentation process

Fresh sludge was taken from a sewage plant in Baotou, and the sludge was transported to the experimental site provided by the Baotou Sludge Disposal Center for pile fermentation immediately after collection; potassium-rich mining waste (200 mesh) was taken from the mining laboratory. The mining waste of potash feldspar, a kind of slate separated from iron core, was obtained from Bayan Obo Mining District; corn straw and corn cob were obtained from a farm near Gumen Town, Tuyu Banner, Baotou City, Inner Mongolia.

In the compost fermentation experiment, each experimental group was mixed according to the ratio shown in Table 1. Three replicate piles were set up in each experimental group, each with a total weight of 200 kg. A number of aeration heads with a total air volume of 7 L/(h·kg) were inserted in each pile, with aeration intervals of 5 min every 25 min, 15 min every 15 min, and no aeration during the warming period, high temperature period, and maturation period, respectively^19^. The pile was turned once a week to ensure the uniformity of the material for 30 days of composting. During the first 10 days of composting, each pile was covered with a light and breathable cotton fabric from 6 pm to 9 am each day for insulation to reduce heat loss due to a sudden temperature drop. Tables 2 and 3 show some physicochemical properties of the raw materials, which were used as a reference for experimental design. Approximately 250 g of compost samples were collected from each pile every three days. The samples were collected from the upper, middle and lower parts of each pile and then mixed into one sample for relevant index testing.

**Table 1:** The material ratio and C/N of each experimental group. (n = 3).

Group	Sewage sludge/%	Potassium-rich mining waste/%	The fermentation materials/% (Maize straw: Corncob = 13: 7)	C/N
DF1	55	25	20	23.25
DF2	60	20	20	22.30
DF3	65	15	20	21.46
CK	80	0	20	19.44

**Table 2:** Physicochemical properties of common composting materials (n = 3).

Parameter	Sewage sludge	Maize straw	Corncob
pH	7.49 ± 0.04	6.41 ± 0.09	6.38 ± 0.05
Total organic carbon (%)	5.25 ± 0.54	42.56 ± 1.54	45.03 ± 0.94
Total N (%)	0.66 ± 0.04	0.77 ± 0.09	0.50 ± 0.03
Organic carbon (%)	5.25 ± 0.35	42.56 ± 2.38	45.03 ± 1.35
Moisture content (%)	78.35 ± 2.17	11.34 ± 1.61	12.44 ± 0.94
C/N	7.95 ± 0.76	55.27 ± 1.54	90.06 ± 2.85
Arsenic (mg·kg^−1^)	10.32 ± 0.90	0.23 ± 0.03	0.38 ± 0.04
Chromium (mg·kg^−1^)	549.07 ± 12.34	3.64 ± 0.34	4.09 ± 0.34
Cadmium (mg·kg^−1^)	7.23 ± 0.34	0.12 ± 0.02	0.13 ± 0.02
Copper (mg·kg^−1^)	189.36 ± 8.92	10.98 ± 1.34	13.80 ± 1.59
Lead (mg·kg^−1^)	186.03 ± 10.24	0.34 ± 0.03	0.13 ± 0.01
Mercury (mg·kg^−1^)	3.12 ± 0.44	0.09 ± 0.01	0.03 ± 0.01

**Table 3:** Physicochemical properties of potassium-rich mining waste. (n = 3).

Parameter	Potash feldspar
pH	8.78 ± 0.26
EC (µs·cm^−1^)	54.54 ± 7.23
Moisture content (%)	0.41 ± 0.12
Total C (%)	0.38 ± 0.06
P (mg·kg^−1^)	389.75 ± 23.75
Ca (mg·kg^−1^)	1378.57 ± 25.64
Mg (mg·kg^−1^)	225.34 ± 7.74
K (g·kg^−1^)	57.32 ± 5.44
Arsenic (mg·kg^−1^)	0.23 ± 0.03
Chromium (mg·kg^−1^)	4.92 ± 0.71
Cadmium (mg·kg^−1^)	0.31 ± 0.03
Copper (mg·kg^−1^)	1.76 ± 0.33
Lead (mg·kg^−1^)	54.84 ± 6.99
Mercury (mg·kg^−1^)	0.38 ± 0.06

### Physical and chemical property analysis

Sample moisture content was determined by oven drying at 105 °C for 6 h. pH and EC were measured by aqueous extract [1:5, sample (w): deionized water (v)]. TN and TOC were determined by a PerkinElmer EA 2400 organic elemental analyzer.

The germination index (GI) was analyzed using watercress seeds according to the standard method^20^ for composting tests. Twenty grams of compost was mixed with 200 ml of distilled water, and after 30 min, the mixture was filtered through a 0.45 mm pore size membrane. Five milliliters of the filtrate was added to Petri dishes with 25 seeds of watercress located on a sheet of filter paper as a support. Three replicates were used for each sample. The same procedure was performed using distilled water instead of compost extracts (control seeds). Plates were placed in a growth chamber at 25 °C for 48 h in the dark. After this period, germination percentage and root lengthening were measured, and GI was calculated based on the following formula:

$$ GI\left( {\text{\% }} \right)\, = \,\left( {\left( {G\% \times L} \right)\,/\,\left( {G_{control} \% \times L_{control} } \right)} \right)\, \times \,{100,} $$

where G % is the germination percentage from seeds exposed to compost extracts, L is the mean root lengthening from seeds exposed to compost extracts, G_control_ % is the germination percentage from control seeds exposed to distilled water, L_control_ and is the mean root lengthening from control samples exposed to distilled water.

The contents of the elements P, Ca, Mg, K, As, Cr, Cd, Cu, Pb, and Hg were analyzed by filtration after digestion with mixed acid (HNO_3_:HF:HClO_4_ = 3:2:1) using ICP‒OES.

### DNA extraction and PCR amplification

Microbial community genomic DNA was extracted from compost samples using the E.Z.N.A.^®^ soil DNA Kit (Omega Biotek, Norcross, GA, U.S.) according to the manufacturer’s instructions. The DNA extract was checked on a 2% agarose gel, and the DNA concentration and purity were determined with a NanoDrop 2000 UV‒vis spectrophotometer (Thermo Scientific, Wilmington, USA). The hypervariable region V3-V4 of the bacterial 16S rRNA gene was amplified with primer pairs 338F (5'-ACTCCTACGGGAGGCAGCAG-3') and 806R (5'-GGACTACHVGGGTWTCTA AT-3'). The hypervariable region ITS1 of the fungal ITS gene was amplified with the primer pair ITS1F (5'-CTTGGTCATTTAGAGGAAGTAA-3') and ITS2R (5'-GCTG CGTTCTTCATCGATGC-3'). PCR amplification instruments were used with an ABI GeneAmp^®^ 9700 PCR Thermocycler (ABI, CA, USA).

### Processing of sequencing data and statistical analyses

The raw 16S rRNA gene sequencing reads were demultiplexed, quality-filtered by fastp version 0.20.0^21^ and merged by FLASH version 1.2.7^22^. Operational taxonomic units (OTUs) with a 97% similarity cutoff^23^^,^^24^ were clustered using UPARSE version 7.1^23^, and chimeric sequences were identified and removed. The taxonomy of each OTU representative sequence was analyzed by RDP Classifier version 2.2^25^ against the 16S rRNA database (Silva v132), ITS database (Unite8.0), and NCBI’s nucleotide database (nr/nt, release 168) using a confidence threshold of 0.7.

Differences in the measured values of the samples are expressed as the mean values. Statistical analysis of the data (test of variance, correlation coefficient) was performed using Python 3.7.4. Sample standard deviations were calculated using Excel 2016. OriginPro2021 software was used to plot the results of the analysis. P values greater than 0.05 were considered nonsignificantly different.

## Results and discussion

### Changes in basic physical and chemical properties

#### Temperature changes in compost

**Figure 1:**
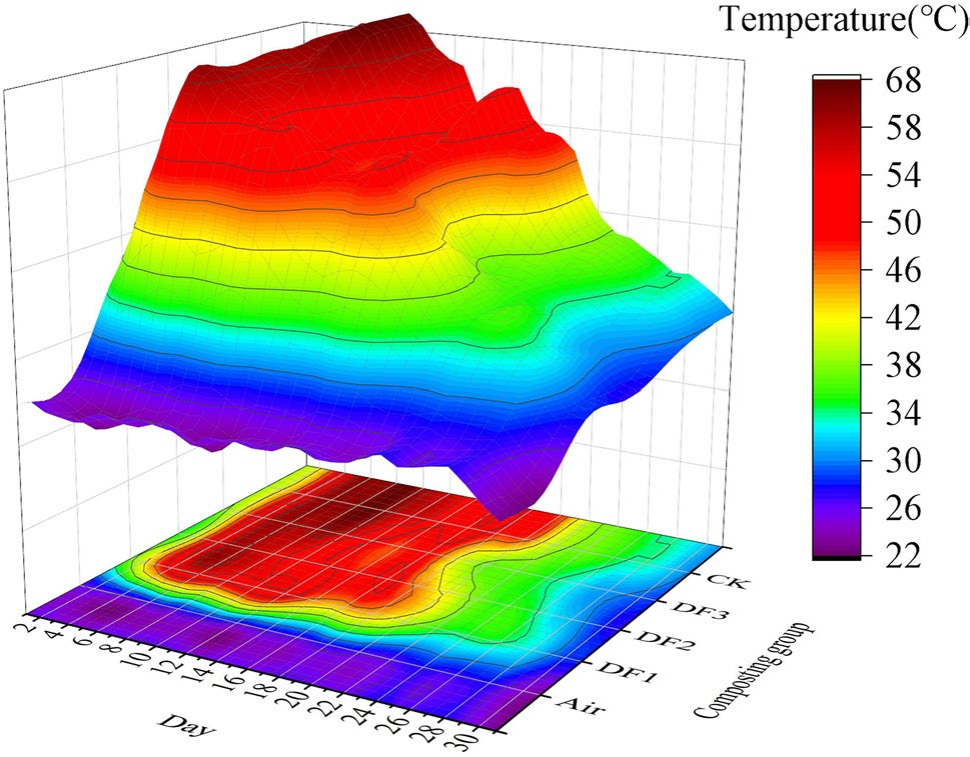
Temperature changes in composting in each experimental group.

The composting temperature reflects both the activity of microorganisms in the composting process and indicates the stage of composting fermentation^26^. The trend of compost temperature was similar in all groups (Fig. 1), and they all entered the high temperature stage (> 50 °C) on Day 4. For the composts with potassium-rich mining waste addition, their core temperatures peaked on Day 6. The experimental group showed peak temperatures 3 days earlier than the control composts without potassium-rich mining waste addition. For composts with different addition ratios, all group peak temperatures were also slightly different. The DF1, DF2, DF3 and CK peak temperatures were 56.5 °C, 58.7 °C, 61.7 °C and 63.7 °C, respectively. The smaller the potassium-rich mining waste addition ratio is, the higher the overall temperature of the composts. This observation was similar to that of Bing Zhao^27^, who suggested that this phenomenon may be due to carbon and nitrogen imbalance. DF1, DF2, DF3, and CK were maintained above 55 °C for 3, 5, 6 and 9 days, respectively. The temperature of DF1 did not meet the Chinese livestock manure harmlessness standard^28^. The core temperature of each compost started to decrease slowly after reaching the peak temperature. At Day 30, the core temperature of each compost was close to room temperature^29^. Ambient temperature is significantly correlated with the core temperature of the compost^30^, and ambient temperature is one of the key factors determining the successful completion of composting^31^. In areas with a large temperature difference between day and night, the low temperature at night will remove a large amount of heat from the compost, and the loss of heat will shorten the duration of the high-temperature phase. At the same time, compost with a relatively large addition of potassium-rich mining waste, which has a large porosity and poor insulation properties, further accelerates the loss of heat. Therefore, the application of potassium-rich mining waste tends to reduce the peak temperature and duration of the high-temperature phase. Ventilation control is directly related to the O_2_ concentration in the compost, and sufficient oxygen can both promote the decomposition of organic waste and reduce GHG emissions^32^^,^^33^. However, the increased frequency of aeration accelerates heat loss and does not favor the duration of the high-temperature period. Balancing the contradiction between aeration and heat loss is one of the key factors in optimizing aerobic composting of minerals and sludge.

#### Change in pH value of compost

**Figure 2:**
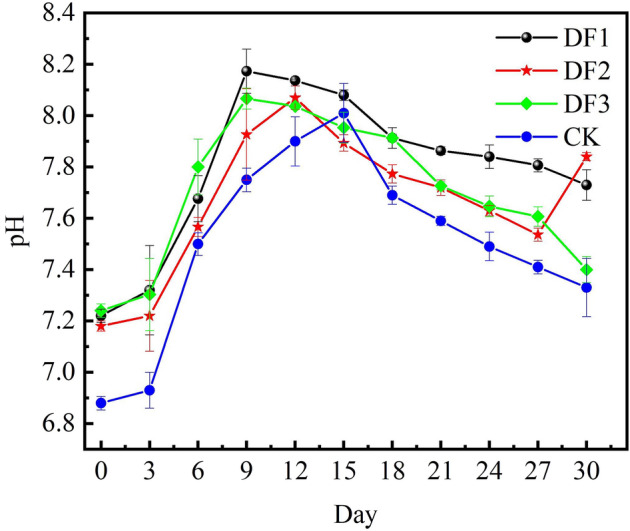
Changes of pH in different days of composting in each group.

pH affects the activity of microorganisms in the composting process, which affects their decomposition rate of organic matter. The pH value can reflect the composting process and final effect to some extent. The addition of potassium-rich mining waste slightly increased the pH of the compost (Fig. 2), but the pH of the compost was at a suitable level throughout the fermentation process in all groups. In the early stage of composting, the mineralization of amino acids, proteins and peptides in the pile led to the accumulation of ammonia nitrogen and the degradation of acidic compounds, resulting in a higher pH^34^. The pH of the compost with potassium-rich mining waste reached the highest value around Day 9, while the control compost without potassium-rich mining waste showed a peak on Day 15. After the peak, the pH of each compost slowly decreased, which may be because the accumulated ammonia nitrogen was involved in the nitrification reaction. In the meantime, ammonia overflowed under turning and aeration as the composting time was extended^35^^,^^36^. On the 30th day, the pH of the compost was stable between 7.33 and 7.84 in all groups.

#### Change in the compost moisture content

**Figure 3:**
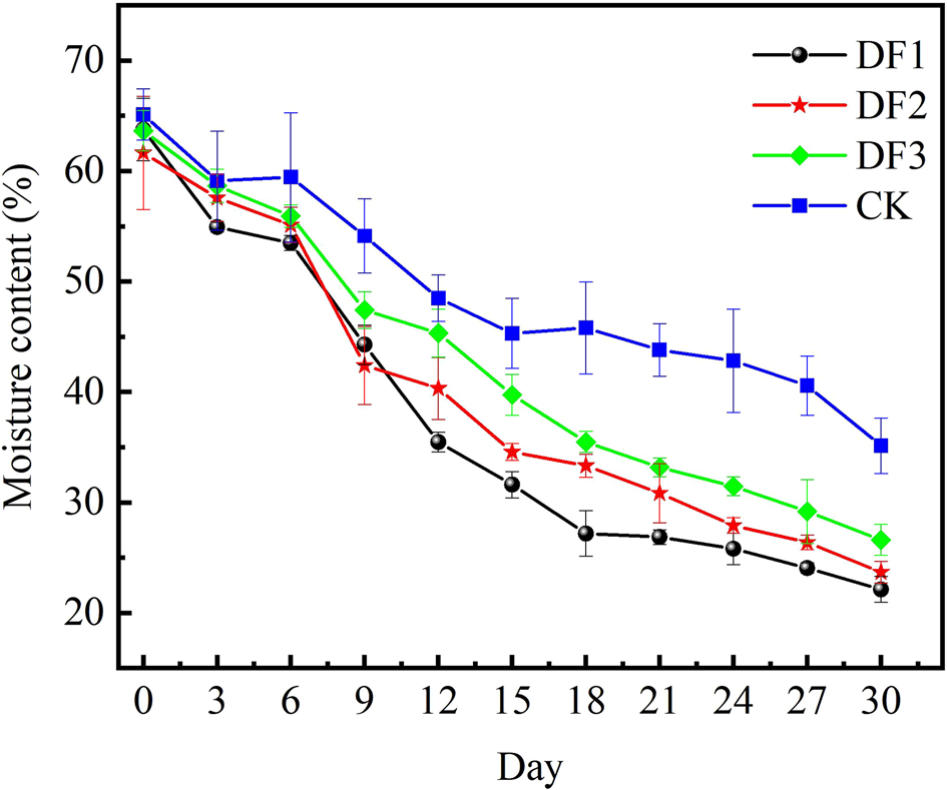
Changes of moisture content in different days of composting in each group.

In addition to the water content of the piles at the initial stage, the decomposition of organic matter by microorganisms during composting also produces water^37^. In the initial warming to high temperature phase, the respiration of microorganisms is stronger, and they decompose organic matter to produce water close to the amount of evaporated water; this phenomenon can be found in Fig. 3. In the warming period and high temperature period, the change in the water content of each compost was relatively flat, and then it started to show a trend of rapid decline. The main reason for the rapid decline in water content was the lower air humidity because the lower air humidity would lead to the compost being dominated by ventilation evaporation and high temperature evaporation. The application of potassium-rich mining waste as a compost additive reduced the percentage of sludge, and the compost became more incompact, so the higher the addition of potassium-rich mining waste, the faster the water content of the compost decreased.

#### Potassium-rich waste minerals accelerated the increase in the germination index

**Figure 4:**
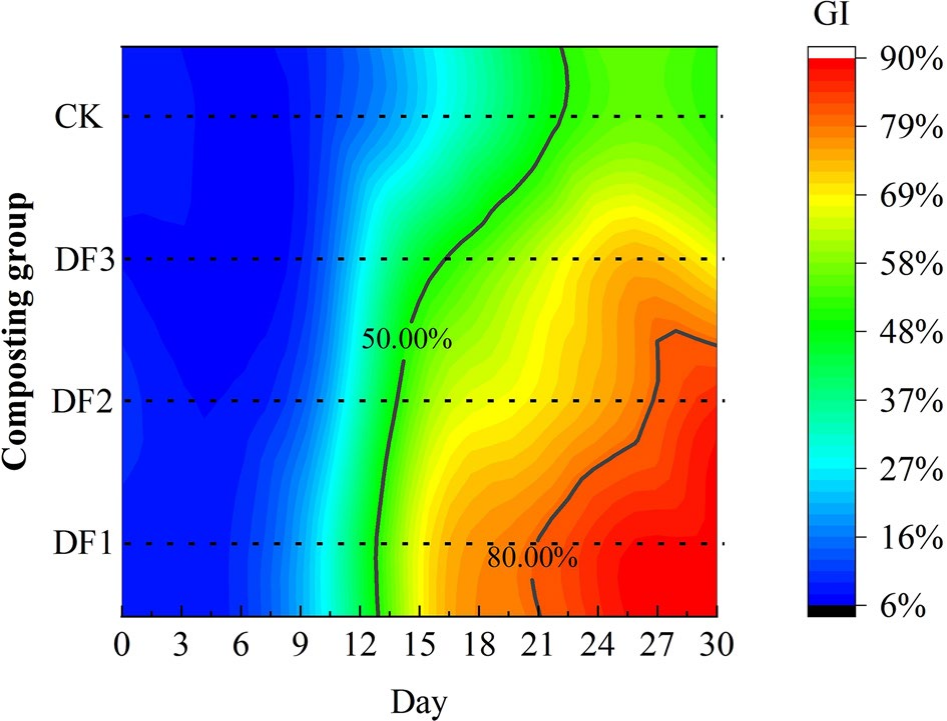
Changes in germination index on different days of composting in each group.

The compost has low or no toxicity to plants when the germination index (GI) is greater than 50%, and the compost is considered to have reached a state of complete decomposition when the germination index is greater than 80%^38^. From Fig. 4, the GI of composts with a higher percentage of potassium-rich waste minerals increased faster. DF1 and DF2 reached complete decomposition (GI > 80%) within 30 days, and DF3 and CK did not meet the condition of complete decomposition within 30 days. The GIs of DF1, DF2 and CK were significantly different (P < 0.05), while DF3 and CK did not show significant differences. The reasonable carbon to nitrogen ratio^39^ and adequate oxygen content provided a more suitable living environment for aerobic bacteria, and the proliferation of aerobic bacteria made the compost fully biodegradable.

### Potassium-rich waste minerals improved the nutrient structure

The addition of potassium-rich mining waste has increased some of the mineral nutrients of the product (Table 4). The content of K_2_O increased by 0.81 ~ 0.83 times, SiO_2_ by 0.20 ~ 0.30 times, MgO by 2.65 ~ 3.3 times and CaO by 0.20 ~ 0.37 times. Mineral elements such as potassium, silicon, magnesium and calcium have greatly increased, and all of these elements have positive effects on the growth of plants^40^^,^^41^. Unlike conventional sludge fertilizers, the mineral elements raised in the product are mainly provided by potassium-rich mining waste, which is a slow-release nutrient and requires organic acids secreted by plants or soil microorganisms to be released. Therefore, this product can alleviate plant health problems caused by imbalances in element content, such as calcium and magnesium ion antagonism^42^, tomato blossom-end rot^43^, and crop collapse. On the other hand, this product also improves the long-term potential of the soil. Therefore, compared with traditional sludge fertilizer, it may have a better conditioning effect on soil lacking the above elements.

**Table 4:** Composition analysis of mature products. (n = 3).

Group	K_2_O (%)	SiO_2_ (%)	MgO (%)	CaO (%)
DF1	5.62 ± 0.88	55.45 ± 2.64	2.45 ± 0.33	2.18 ± 0.23
DF2	5.61 ± 0.79	53.76 ± 4.32	2.08 ± 0.39	1.94 ± 0.08
DF3	5.66 ± 0.69	51.23 ± 4.93	2.12 ± 0.21	1.91 ± 0.16
CK	3.09 ± 0.71	42.64 ± 4.22	0.57 ± 0.18	1.59 ± 0.17

### Composition and succession of the microbial community

#### Analysis of intergroup differences between bacteria and fungi

**Figure 5:**
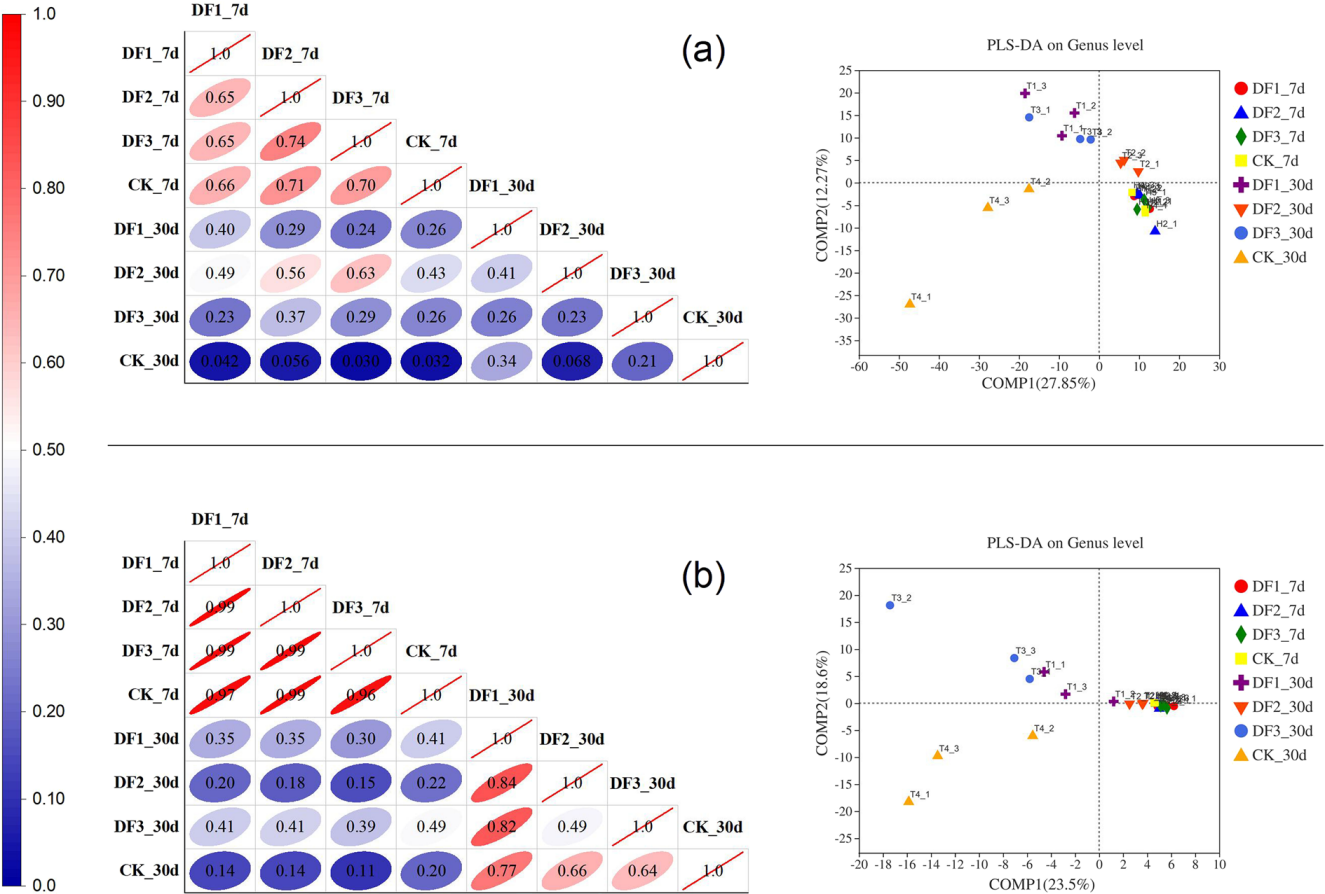
Pearson correlation matrix analysis (n = 978) and partial least squares discriminant analysis (PLS-DA) of compost about the bacteria (**a**) and fungus (**b**). DF1_7d, DF2_7d, DF3_7d and CK_7d represent the samples on Day 7; DF1_30d, DF2_30d, DF3_30d and CK_30d represent samples on Day 30.

To visually describe the species differences between samples, the differences in bacterial and fungal communities were analyzed by using the Pearson correlation matrix and partial least squares discriminant analysis (PLS-DA). The species distance between compost samples in the high-temperature period was more concentrated (Fig. 5a), and the Pearson correlation matrix also showed a high correlation (0.65 ≤ r ≤ 0.74). This was consistent with the expected results because the bacterial community structure screened by the high-temperature environment resulted in a high similarity. These data indicated that potassium-rich mining waste did not show a significant effect on the core bacterial community in the high-temperature period. The PLS-DA plots for fungi showed similar results to those for bacteria (Fig. 5b). However, compared to bacteria, the Pearson correlation matrix for fungi showed higher correlations during the decay period (0.49 ≤ r ≤ 0.84) and much higher correlation coefficients during the high temperature period than for bacteria. This result implied that the application of potassium-rich mining waste had much less of an effect on the fungal community than on the bacterial community.

#### Microbial diversity index

**Figure 6:**
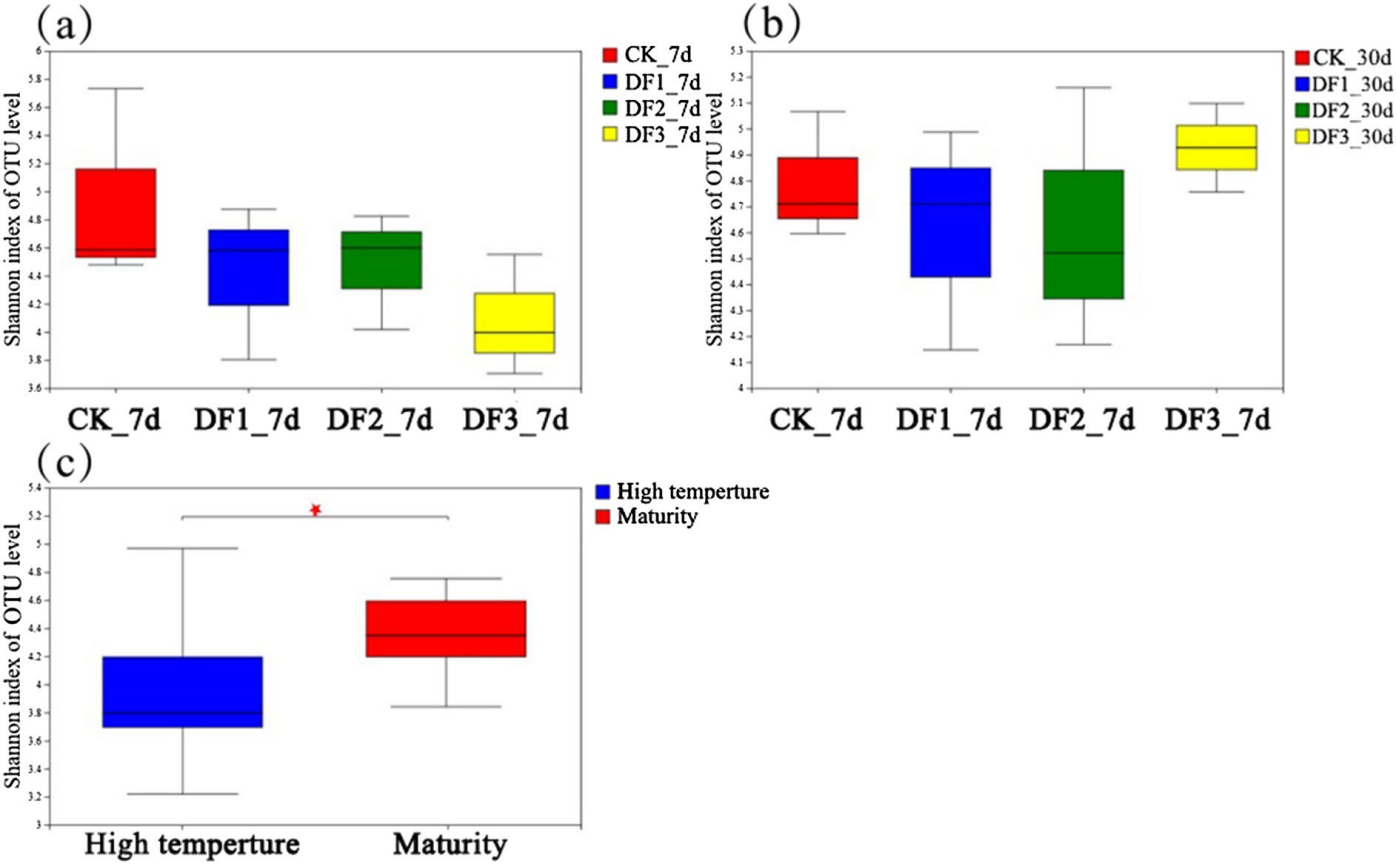
Shannon diversity index analysis. DF1_7d, DF2_7d, DF3_7d and CK_7d represent the samples on Day 7; DF1_30d, DF2_30d, DF3_30d and CK_30d represent samples on Day 30. High temperature and maturity represent high temperature and maturity stage, respectively. “*” represents p < 0.05 is a significant difference.

The Shannon index is commonly used to characterize the level of microbial diversity, and the higher the value is, the higher the level of diversity of microbial communities in the sample. As shown in Fig. 6, the Shannon indices of the four pile groups were CK control, DF2, DF1, and DF3 in descending order during the high temperature period; the Shannon indices of the DF1 and DF2 piles were close to each other, but the overall Shannon indices did not show statistically significant differences. This is consistent with the studies of^44^ and^45^, in which the high temperature killed a large number of bacteria, resulting in a lower Shannon index, which reflected that the species were more homogeneous, with some thermophilic bacteria dominating and having a better effect on the decomposition of organic matter.

#### Phylogenetic relationship and community composition of thermophilic bacteria at the high-temperature stage

**Figure 7:**
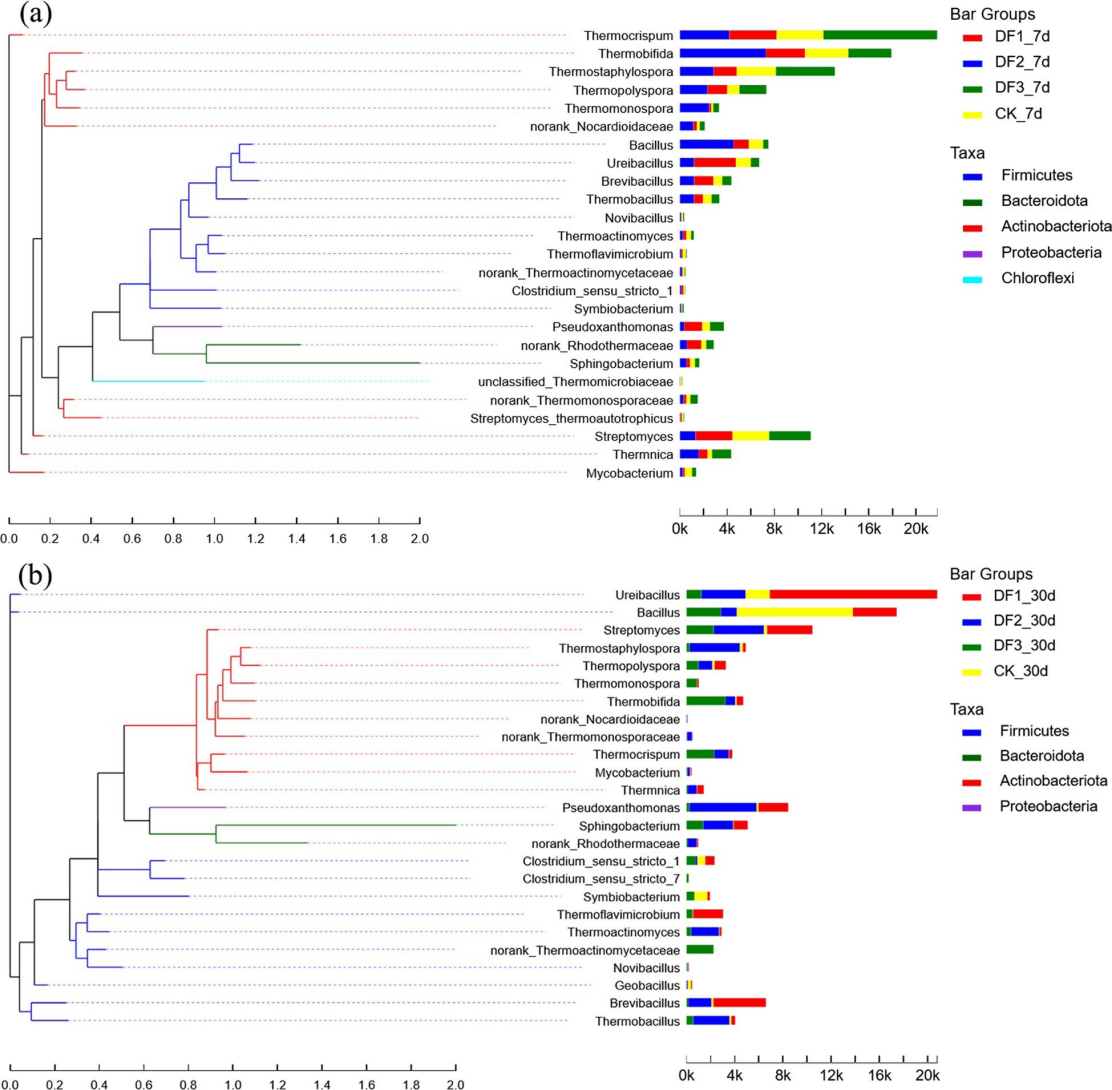
Phylogenetic tree of thermophilic bacteria during the high-temperature (**a**) and decomposition stages (**b**).

The thermophilic bacterial communities were very rich during the aerobic composting processes, and 49 different strains were detected in this experiment (Fig. 7). The abundance of thermophilic bacteria was different in each group, but it did not affect the analysis of one composting period. The genus abundance of the high-temperature stage was in descending order of Thermocrispum, Thermobifida, Thermostaphylospora, Streptomyces and Bacillus. Thermocrispum is an aerobic bacterium that grows at temperatures between 20 and 62.5 °C, which is almost consistent with the peak temperature of this experiment, so it is not surprising that it dominates the absolute abundance of composting during the high temperature period^46^. Thermobifida is commonly found in manure aerobic composting^47^ purified carboxy methyl cellulase from Thermobifida Q-0, which retains high activity above 60 °C in a weakly alkaline environment. The weakly alkaline environment due to potassium-rich waste minerals exactly meets the pH conditions for this enzyme. Thermocrispum, Thermobifida, Thermostaphylospora and Streptomyces are closely related to Actinobacteriota. Bacillus, Ureibacillus, Brevibacillus and Thermobacillus are closely related to Firmicutes. The synergistic action of Thermobacillus and some other bacteria promoted the decomposition of hemicellulose. When compost was transferred to maturity, due to the decrease in temperature, thermophilic bacteria with limited temperature tolerance took over the dominance of thermophilic bacteria in the high temperature period, particularly Ureibacillus, Bacillus, Streptomyces, Pseudoxanthomonas and Brevibacillus. Pseudoxanthomonas mainly uses lignin as a carbon source^48^, and the mature stage leaves a large amount of undecomposed lignin, which improves the nutrient advantage of Pseudoxanthomonas.

#### Functional classification of thermophilic bacteria during high temperature

**Figure 8:**
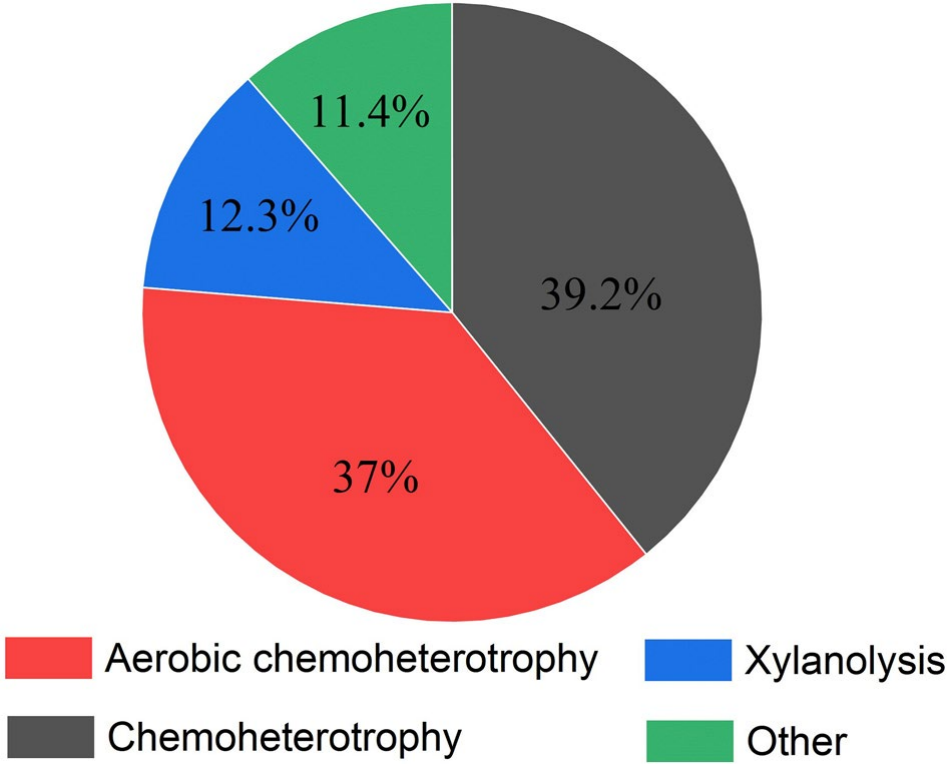
FAPROTAX abundance of thermophilic bacteria in aerobic composting.

FAPROTAX maps prokaryotic taxa to metabolic or other ecologically relevant functions and is highly informative for composting studies. From the results of the functional prediction analysis of the thermophilic bacterium FAPROTAX (Fig. 8). Thermophiles are mainly divided into aerobic chemoheterotrophy, chemoheterotrophy, xylanolysis and others. The proportions of aerobic chemoheterotrophy and chemoheterotrophy are 37% and 39.2%, respectively. The data reflect the importance of oxygen to thermophiles.

#### Potassium-rich mining wastes can promote the propagation of thermophilic bacteria by increasing air infiltration

**Figure 9:**
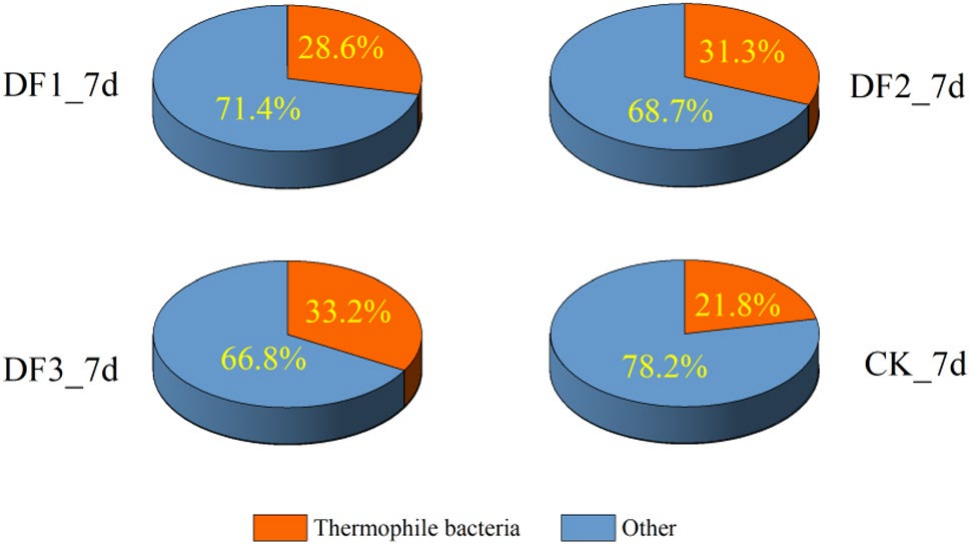
Percentage of thermophilic bacteria in each sample during high temperature period.

**Figure 10:**
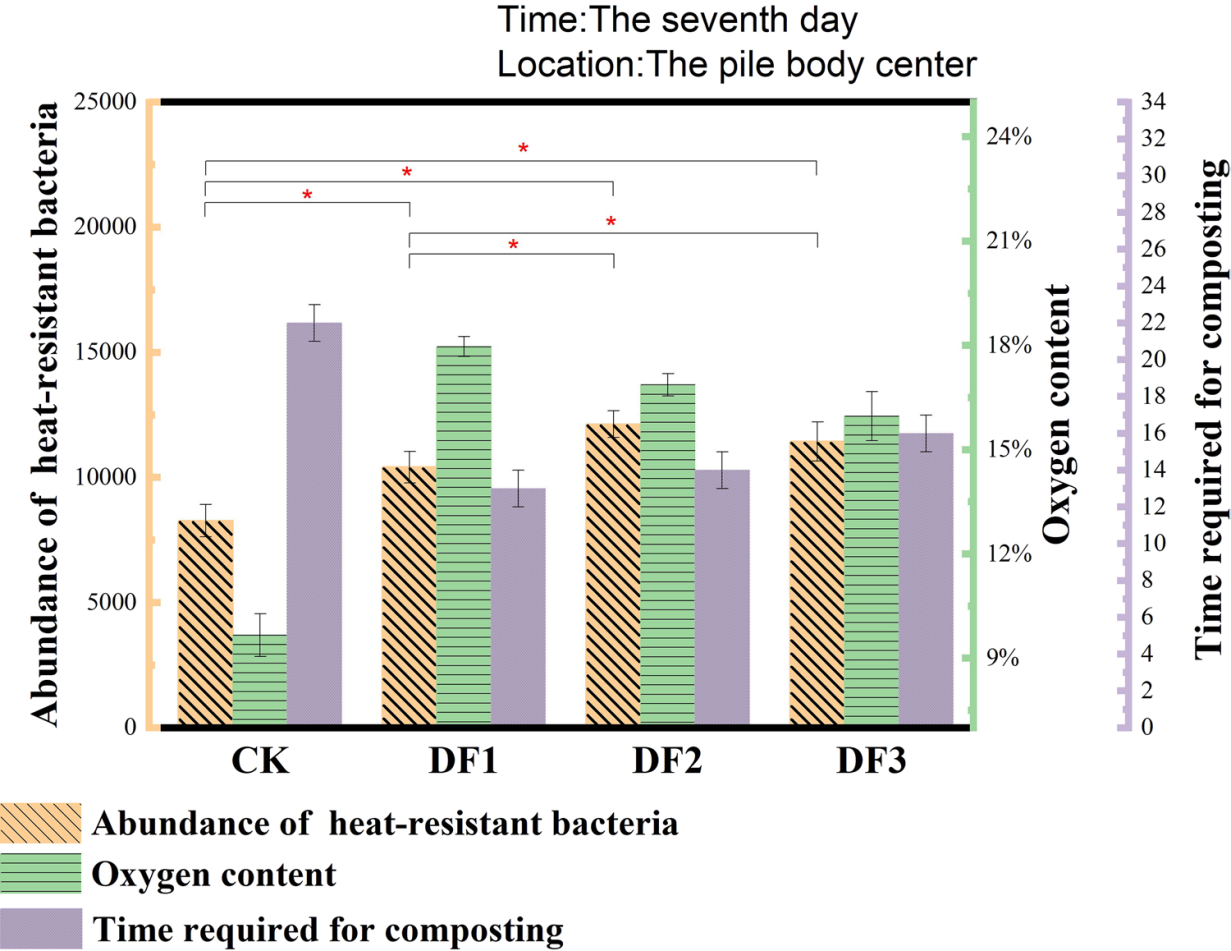
The abundance of thermophilic bacteria, oxygen content and the time required for composting; “*” means that p < 0.05 indicates a significant difference.

Thermophilic bacteria occupy an important position in the high-temperature aerobic composting process, and they largely determine the degradation efficiency of organic waste in the high-temperature phase^49^. Common genera of thermophilic bacteria in aerobic composting include but are not limited to *Brevibacillus*^50^, *Thermomonospora*, *Thermobacillus*, *Pseudoxanthomona*^51^, *Geobacillus*^52^, *Thermocrispum*, *Ureibacillus*^53^, *Thermobifida*, *Thermostaphylospora*, *Thermopolyspora*, *Thermotunica*, *Mycobacterium*, *Thermoflavimicrobium*, *Thermoactinomyces*, *Novibacillus*, *Symbiobacterium*, *Schlegelella*, *Thermobispora*, and *Ruminiclostridium*. The statistical thermophilic bacteria varied in each group (Fig. 9), with potassium-rich waste minerals making the percentage of thermophilic bacteria between 28.6% and 33.2% and only 21.8% for CK. The abundance of thermophilic bacteria was analyzed in combination with the data of the oxygen content and the days required for decomposition (Fig. 10). The abundance of thermophilic bacteria in the experimental group was significantly higher than that in the control group. The control group hypoxia degree was more obvious. A large proportion of thermophiles are aerobic and partly aerobic bacteria, and a low oxygen content will affect cell metabolism or even lead to death, so the abundance of thermophilic bacteria in composts and oxygen content usually show a positive correlation. The days required for decomposition of the DF1, DF2 and DF3 composts were 9, 8 and 6 days earlier than those of the control group, respectively. The conclusion obtained from the above analysis is that the addition of potassium-rich mining waste reduced the specific gravity of sludge, and potassium-rich waste minerals (200 purposes) adhered to the surface of the sludge, making them more dispersed. These factors increased the ability of air to penetrate into the compost. The high oxygen content promoted the reproduction of thermophilic bacteria in the high temperature period, further improving the compost degradation efficiency, which in turn shortened the time required for compost decomposition.

## Conclusion

As the mineral additive of sludge aerobic composting, potassium-rich mining waste can raise the air intrusion capacity, which promotes the reproduction of aerobic thermophilic bacteria and shortens the sludge composting cycle. However, it has the disadvantage of a lower high-temperature duration and peak temperature value, resulting in a lower sterilization rate and limiting its large-scale promotion. Therefore, the amount of potassium-rich waste minerals should not be higher than 20%.

## Data Availability

All data generated or analyzed during this study are included in this published article [https://doi.org/10.6084/m9.figshare.21432768].
